# Eurythmy therapy versus slow movement fitness in the treatment of fatigue in metastatic breast cancer patients: study protocol for a randomized controlled trial

**DOI:** 10.1186/s13063-020-04542-5

**Published:** 2020-07-06

**Authors:** Delphine Meier-Girard, Karin Ribi, Gisa Gerstenberg, Thomas Ruhstaller, Ursula Wolf

**Affiliations:** 1 Institute of Complementary and Integrative Medicine, University of Bern, Fabrikstrasse 8, 3012 Bern, Switzerland; 2 International Breast Cancer Study Group, Coordinating Center, Bern, Switzerland; 3 Breast Center of Eastern Switzerland, St. Gallen, Switzerland

**Keywords:** Cancer-related fatigue, Eurythmy therapy, Mindful therapies, Complementary therapies, Metastatic breast cancer, Randomized controlled trial

## Abstract

**Background:**

Cancer-related fatigue (CRF) is the most taxing symptom for many breast cancer patients during and after therapy. In patients with metastatic disease, the prevalence of CRF exceeds 75%. Currently, there is no gold standard for the treatment of CRF. Physical activity can reduce CRF and is recommended during and after cancer treatment, but may be too burdensome for patients with metastatic breast cancer. The aim of this study is to assess the effect on fatigue of eurythmy therapy (ERYT) compared to slow movement fitness (CoordiFit) in metastatic breast cancer patients.

**Methods:**

The ERYT/CoordiFit study is a randomized controlled, open-label, two-arm, multi-center Swiss clinical trial. A sample of 196 patients presenting with CRF will be recruited by oncologists from the departments of clinical oncology at each local study site. All participants will be randomly allocated to the intervention or control group in a 1:1 ratio. The control group is an active control intervention (CoordiFit) in order to control for potential non-intended effects such as therapist-patient interaction and participation in a program. Both ERYT and CoordiFit exercises are easy to learn, and the training sessions will follow the same frequency and duration schedule, i.e., 13 standardized therapy sessions of 45 min (once a week for 6 weeks and then once every second week) during the total intervention period of 20 weeks. The primary endpoint of the study is the change from baseline over the whole intervention period (i.e., including measurements at baseline, weeks 8, 14, and 20) in the Functional Assessment of Chronic Illness Therapy – Fatigue (FACIT-F) subscale score.

**Discussion:**

This study is the first-known randomized clinical trial assessing eurythmy therapy in the treatment of fatigue in metastatic breast cancer patients. Given the distress that fatigue causes patients, it is important to validate treatment options. If eurythmy therapy proves beneficial in CRF as part of this randomized controlled clinical trial, the study may be very impactful with implications not only for metastatic breast cancer patients but also for other cancer patients, health care personnel, scientists, and funding and regulatory bodies.

**Trial registration:**

The ERYT/CoordiFit trial was registered at the US National Institutes of Health (ClinicalTrials.gov) on July 18, 2019, #NCT04024267, and in the portal for human research in Switzerland on December 3, 2019, #SNCTP000003525.

## Introduction

Cancer-related fatigue (CRF) is the most taxing symptom for many breast cancer patients [1]. Depending on the methods used for measuring fatigue, patient group characteristics 15 to 99% of breast cancer patients experience fatigue during therapy. Also, 25 to 30% of patients continue to experience fatigue up to 5 years after completion of treatment or possibly even longer [2]. In patients with metastatic disease, the prevalence of CRF exceeds 75% [3, 4]. CRF has been shown to be the symptom that has the largest impact on quality of life [5], with negative effects on work, social activities, and daily activities [6–9]. Furthermore, CRF might lead to treatment discontinuation [2].

The pathophysiological mechanism behind CRF has not been clarified yet and is considered to be multifactorial [2, 10].

Currently, there is no gold standard for the treatment of CRF [2, 11]. The National Comprehensive Cancer Network (NCCN) Guidelines [3, 12], as well as the American Society of Clinical Oncology [13], recommend a combination of approaches. These guidelines and a recent meta-analysis [14] highlight psychosocial interventions (i.e., including education and counseling of patient and family) and physical activity as having the strongest evidence base for treating fatigue during and after cancer treatment. However, physical activity may in itself be too burdensome for metastatic breast cancer patients due to metastases or other comorbid illnesses [3]. Pharmacological treatment, including therapy with antidepressants or hematopoietic growth factors, is not recommended [2, 15]. There is increasing evidence that psychostimulants, in particular methylphenidate, are likely to benefit patients with CRF [15], but the use of pharmacological agents remains controversial [2, 3]. Finally, there is some evidence that non-pharmacological mind-body techniques (e.g., tai chi) may be effective in reducing CRF [2, 3, 11, 16–26]. So far, the available data are not sufficiently reliable to formulate final recommendations [27]. Further studies are needed to confirm the effectiveness of such interventions. Given the prevalence of CRF and the distress it causes to patients, work to advance new options to improve fatigue is highly desirable.

Eurythmy therapy has been used for more than 80 years. Eurythmy essentially consists of converting the sounds of language into movements that regulate and harmonize healing processes and thus foster recovery and regaining strength. A key aspect of the eurythmy therapy is its role in training awareness and self-perception [28–32], thus inducing physiological and therapeutic effects. Eurythmy therapy exercises are specific for a distinct medical indication, symptom, or disease [32]. In particular, for breast cancer patients, the so-called cancer series “O-E-M-L-I-B-D” is applied [26, 32].

In cancer patients, the demand for mind-body therapies has increased during recent years [11, 33–38], and breast cancer patients are more likely to use any complementary health approach than patients with other cancers [35, 39]. Eurythmy therapy has specific effects in healthy subjects, such as reducing fatigue [33] and stress [40, 41]. Moreover, studies describe a reduction of a range of symptoms [42–48], as well as improvements in quality of life [40, 48] in patients with various chronic diseases and in different target groups (i.e., children, adolescents, adults) [49, 50]. As observed with other mind-body therapies [51–55], improvements have also been described in heart rate variability (HRV) parameters (i.e., parameters that measure the adaptability of the autonomic nervous system), as well as in circadian patterns of HRV, after eurythmy therapy training [33, 56–59]. This improvement in HRV parameters relates to a change in autonomic regulation toward a more relaxed pattern and an improvement of complexity (i.e., adaptability) in the heart rate dynamics. These results suggest that eurythmy therapy might have positive effects on the autonomic nervous system. The improved relaxation may lead to better stress-coping strategies, a better circadian rhythm, and ultimately to improved quality of life. Studies generally report good compliance and satisfaction of participants and patients with eurythmy therapy [40, 50]. Finally, eurythmy therapy has been used in adults and children for several decades without any harmful effects [33, 49, 50].

The aim of this study is to assess the effect of eurythmy therapy on fatigue in metastatic breast cancer patients as compared to slow movement fitness.

## Methods/design

### Study design

The ERYT/CoordiFit study is a randomized controlled, open-label, two-arm, multi-center superiority trial. A sample of 196 female patients with metastatic breast cancer presenting with CRF will be randomly allocated to the intervention or control group in a 1:1 ratio. The control group is an active control intervention (CoordiFit) in order to control for unintended effects such as the therapist-patient interaction and active engagement (being part of a program). It consists of slow, standardized exercises that address physical coordination, stability, balance, and dexterity. These exercises are non-specific with respect to CRF and do not include mindfulness features. The study is planned to be conducted in 14 centers across Switzerland.

### Participants and recruitment

The participants will be recruited by oncologists from the departments of clinical oncology at each local study site. Our study will be advertised on the homepage of the Institute for Complementary and Integrative Medicine and flyers will be available at each study site. Subjects are only included if they meet a pre-defined FACIT-F subscale score. To avoid bothering subjects by fully informing them about a study for which they prove ultimately ineligible, a two-step consenting procedure is implemented:

1. Each potential participant will first be asked to sign a short “preliminary consent” form for completion of the FACIT-F questionnaire. The oncologist or a study nurse will explain to the patient briefly the nature of the study and its purpose and inform her that she might be eligible for the study depending on her FACIT-F subscale score. The preliminary consent form signed by the patient and by the oncologist or the study nurse will be documented in the patient’s source documentation.
2. Eligible patients will then be offered to participate in the study and, if interested, will be fully informed about the trial using the full patient information and informed consent form. Written informed consent will be obtained before the participant is submitted to any study procedure. The patient has at least 24 h to consider whether to participate and can subsequently send back the signed consent form by post to the study site (a stamped, addressed envelope will be provided). A study nurse will contact the patient by phone after 6 working days if a patient does not send back the consent form. Information on the schedule of study procedures and assessments are shown in Table 1.

**Table 1:** Schedule of enrollment, interventions, and assessments

Study period	Screening	Baseline	Intervention													Follow-up	
Time	14–1 days prior to baseline	0 (14–0 days prior to first training session)	Week 1	Week 2	Week 3	Week 4	Week 5	Week 6	Week 8	Week 10	Week 12	Week 14	Week 16	Week 18	Week 20	+ 6 months after week 20	+ 12 months after week 20
**Enrolment**																	
Preliminary informed consent for FACIT-F administration	X																
Eligibility screen	X																
Informed consent	X																
Randomization		X															
**Intervention**																	
ERYT/CoordiFit sessions			1	2	3	4	5	6	7	8	9	10	11	12	13		
**Assessments**																	
Medical history and treatments		X													X		
Patient’s Baseline questionnaire		X															
FACIT-F	X^1^								X			X			X	X	X
PSQI		X							X			X			X	X	X
PHQ-9 and GAD-7		X													X	X	X
BPI		X							X			X			X	X	X
PRFS		X													X	X	X
NCCN Distress Thermometer		X							X			X			X	X	X
Mobility of the arm		X							X			X			X	X	X
Therapist’s Questionnaire			X	X	X	X	X	X	X	X	X	X	X	X	X		
Patient diary			Every day														
Patient’s Last Session questionnaire															X		
Patient’s Follow-up questionnaire																X	X
End of Study questionnaire																	X

*BPI* Brief Pain Inventory, *FACIT-F* Functional Assessment of Chronic Illness Therapy – Fatigue, *NCCN* National Comprehensive Cancer Network, *PRFS* Patient-Reported Functional Status, *PHQ* Patient health questionnaire, *PSQI* Pittsburgh Sleep Quality Index

^1^The patient will be asked to fill in the FACIT-F questionnaire only after he returns the signed preliminary informed consent

### Inclusion and exclusion criteria for the trial

Patients who meet the following criteria will be deemed eligible for the trial: (a) female aged 18 years or older, (b) CRF according to the FACIT-F (FACIT-F subscale score < 34 [60]), (c) histologically or cytologically confirmed metastatic breast cancer, (d) Eastern Cooperative Oncology Group (ECOG) grade 0 or 1, (e) ability to physically and cognitively perform an active movement therapy, (f) ability to provide informed consent as documented by signature, and (g) ability to read, write, and speak German, French, or Italian.

Patients who meet the following criteria will be excluded from the trial: (a) inability or contraindication that would prevent prolonged follow-up, or to undergo the investigated intervention or control intervention, in the opinion of the investigator; (b) patients with psychiatric, addictive or any disorder that prevents the patient from adhering to the protocol requirements, in the opinion of the investigator; (c) significant uncontrolled cardiac disease (e.g., unstable angina, recent myocardial infarction); and (d) hemoglobin < 9 g/dl.

During the trial, all participants will continue to receive the usual care provided by their health care providers. Cancer-specific medications/treatments (e.g., chemotherapy or radiation therapy, surgery, immunotherapy, endocrine therapy) received before or during the study are documented and examined later in data analyses, but not restricted.

### Randomization

Randomization procedures were implemented at the Clinical Trial Unit of the University of Bern (CTU Bern) according to dedicated standard operating procedures. Allocation will be done by a study nurse via a dedicated website within the clinical trial management system REDCap, which also contains the electronic case report forms. All participants will be randomly allocated to the intervention or control group in a 1:1 ratio. Randomization will be stratified by center using permuted blocks with random block sizes. Only system administrators who are otherwise not involved in the trial will have access to the algorithm and stored lists during the recruitment period. The underlying randomization lists and details of the algorithm will not be disclosed but kept securely at CTU Bern. All these measures will help ensure concealment of allocation.

### Endpoints

The primary endpoint of the study is the change from baseline over the whole intervention period (i.e., including measurements at baseline, weeks 8, 14, and 20) in the Functional Assessment of Chronic Illness Therapy – Fatigue (FACIT-F) subscale.

Secondary endpoints are the following:

- The change from baseline to the end of intervention (i.e., including measurements at baseline and at week 20) in the FACIT-F subscale
- The change from baseline over the whole intervention period (i.e., including measurements at baseline, weeks 8, 14, and 20) in the FACT-G, Pittsburgh Sleep Quality Index (PSQI), Brief Pain Inventory (BPI), Patient Health Questionnaire-9 (PHQ-9), General Anxiety Disorder-7 (GAD-7), NCCN Distress Thermometer, and arm mobility scores.
- Number of patients who return to work during the intervention phase or the follow-up.
- Retention will be measured by the number, period to, and reasons for attrition for each group. Compliance will be measured by the number of sessions attended, and the number of hours of home training per week.
- Targeted quantitative metabolomics will be investigated in 30 patients as a translational research element. Analysis of amino acids, organic acid profiles, and acylcarnitine profiles in serum and urine will be measured with high-performance liquid chromatography and liquid chromatography-mass spectrometry, allowing for analysis of over 100 metabolites. Metabolomic data will be measured at baseline and at the end of intervention (week 20).

Exploratory endpoints of the study are follow-up measurements (6- and 12-month follow-up from week 20) in FACIT-F subscale, FACT-G, NCCN Distress Thermometer, PSQI, BPI, PHQ-9, GAD-7, and arm mobility.

### Outcome measures

Fatigue and quality of life are measured using the FACIT-F. The FACIT-F belongs to the FACIT family of questionnaires developed by the original Functional Assessment of Cancer Therapy – General (FACT-G) [61, 62]. FACT-G is a patient-reported outcome measure used to assess health-related quality of life in patients undergoing cancer therapy. It assesses the impacts of cancer therapy in four primary quality of life domains: physical, social/family, emotional, and functional well-being. The FACT measurement system is a widely used measure of patient-reported outcomes in cancer research [63]. FACIT-F consists of the FACT-G plus the FACIT-F subscale comprising 13 fatigue-related items, with a total of 41 items [64]. The FACIT-F subscale assesses self-reported fatigue and its impact on daily activities and function. It has demonstrated reliability, validity, and responsiveness to change. The questionnaire is easy to read and quick to complete (about 10 min).

Sleep quality is measured using the PSQI. The PSQI is a self-rated questionnaire which assesses sleep quality and disturbances in the past 4 weeks [65]. It is currently the only standardized clinical instrument that covers a broad range of indicators relevant to sleep quality [66]. The questionnaire has shown strong reliability and moderate structural validity [66]. PSQI consists of 19 items divided into 7 component scores: subjective sleep quality, sleep latency, sleep duration, habitual sleep efficiency, sleep disturbances, use of sleeping medication, and daytime dysfunction. The sum of scores for these seven components yields one global score ranging from 0 to 21 [65]. A global score ≤ 5 is associated with good sleep quality, while a score > 5 is associated with poor sleep quality. The questionnaire is easy to complete within 5 to 10 min.

Anxiety and depression will be measured using the GAD-7 (7 items) and the PHQ-9 (9 items), respectively. Both instruments have evidence of reliability and validity [67–69].

Pain is measured using the BPI. The BPI is a 9-item self-report measure that assesses worst, least, and average levels of pain and the degree to which pain interferes in activities, mood, relationships, sleep, and enjoyment of life [70]. This measure is widely used with cancer patients and has evidence of reliability and validity [70, 71].

Patient distress is measured using the NCCN Distress Thermometer. The NCCN Distress Thermometer measures distress on a scale of zero to 10, 10 being the worst [72]. The NCCN Problem List allows patients to inform their doctor if they are having concerns in areas such as practical, family, emotional, spiritual, and physical problems. This list will not be used as part of this study in order to limit the number of questions.

The functional status of the patient is measured using the Patient-Reported Functional Status (PRFS). The PRFS is a patient-completed measure of performance status. It is the activities/function domain of the Patient-Generated Subjective Global Assessment (PG-SGA), which is a widely-used, well-validated measure used in patients with cancer [73, 74].

Factors including a patient’s socio-demographic data, time since diagnosis, stage of disease, initial metastatic breast cancer or later development, previous and current cancer therapy, co-morbidities which may cause CRF, and level of physical activity (at least once a week) are collected through tailored electronic questionnaires (medical history and treatments questionnaires and patient questionnaires) and will be taken into account for the evaluation of the effect of intervention on CRF.

### Study interventions

Both ERYT and CoordiFit exercises are easy to learn, and the training sessions will follow the same frequency and duration schedule, i.e., 13 standardized therapy sessions of 45 min (once a week for 6 weeks and then once every second week) during the total intervention period of 20 weeks. Patients are instructed by a eurythmy therapist or physical therapist, according to the group of allocation, in sessions with 1 to 4 patients. Therapists are available in all areas of Switzerland, either affiliated with institutions (e.g., hospitals) or work in private practices. Patients are asked to additionally train at home 15 min per day, 6 times a week, and compliance of the home training is reported by the patient in a diary.

All eurythmy and physical therapists will participate in an introductory teaching session before the start of the study, to familiarize themselves with the study specifics.

### Experimental group: ERYT

The well-established cancer series “O-E-M-L-I-B-D” that is specific and standardized for breast cancer patients and which has been applied in breast cancer patients for decades will also be applied here [26, 32]. Through this indication-specific concept, the eurythmy therapy distinguishes itself from other mindful methods (e.g., tai chi). In Switzerland, eurythmy therapy is usually prescribed by physicians. Patients are treated by certified eurythmy therapists who have successfully completed a standardized 6 years of training, have passed a federal examination, and hold a federal diploma. For patients, eurythmy therapy is easy to learn and can be practiced anywhere (e.g., at home, at work, while traveling) and irrespectively of an individual medical condition or state of well-being. Eurythmy therapy also empowers patients by providing a tool that supports them in being active and self-reliant. The practices will be introduced using a standard progression from simpler to more challenging movements over the course of the intervention depending on the patients’ capabilities. Patients can perform and maintain the postures without stress and tension.

### Active control intervention: CoordiFit

The CoordiFit program consists of slow, standardized exercises that address physical coordination, stability, balance, and dexterity. These exercises serve as a control intervention and are non-specific with respect to CRF and breast cancer. They mimic the exercises of ERYT but have no mindfulness features. The exercises in the control group focus on movements such as mobilization of the backbone (roll down with the head toward the feet, vertebra by vertebra, coming back up, lastly with the head), the shoulders (roll shoulders, swing arms), legs (tap with one foot in front, sideward, and backwards, walking with tapping: coordinate the rhythm with the tap in a walking movement), balancing (standing and rocking on toes, positioning the arms, torso, and one leg parallel to the floor), and coordination exercises (clapping and tapping with hands and feet, respectively in different orders).

### Procedures

#### Screening (14–1 day(s) prior to allocation)

After providing the signed preliminary informed consent for completion of the FACIT-F questionnaire, the patient will be asked to fill in a printed version of the FACIT-F questionnaire in order to determine whether she is eligible for the study. The study nurse will enter the patient’s answers into the dedicated study database (REDCap). If the FACIT score is < 34, the patient will be offered a place on the study.

#### Allocation and baseline (14–0 day(s) prior to first training session)

After obtaining the patient’s signed informed consent, the patient will be randomized and the study nurse will fill in the Medical History and Treatments questionnaire in REDCap. The study nurse will contact the patient to specify which group she has been allocated to and will answer any questions the patient may have. Then, the study nurse will contact the therapist according to the group and area of the patient and ask her to call the patient to arrange the first training session. The patient will receive via email a link (generated by REDCap) to open in a web browser the questionnaires to be filled in (i.e., Patient’s Baseline questionnaire, PSQI, PHQ-9, GAD-7, BPI, NCCN Distress Thermometer, arm mobility, and PRFS). These questionnaires constitute the baseline assessment and consequently must be filled in before the first session.

#### Session 1 (week 1)–session 13 (week 20): intervention, assessment tools/questionnaires, and patient diary

During the intervention phase, the patient will perform 13 sessions of ERYT or CoordiFit. During the whole intervention phase, regular physical activity (at least once a week) and compliance with home training will be reported by the patient in a pre-printed diary which will be provided to the patient. The patient will send back the diary by postal mail after the last session (addressed and stamped envelope will be provided by the study site). At each session, the eurythmy or physical therapist will record in REDCap whether the patient attended, the reason if not, and if the patient was able to execute all movements (therapist’s questionnaire).

#### Sessions 7 and 10 (weeks 8 and 14): assessment tools/questionnaires

At weeks 8 and 14, the patient will receive via email a link (generated by REDCap) to open in a web browser the questionnaires to be filled in (FACIT-F, PSQI, PHQ-9, GAD-7, BPI, NCCN Distress Thermometer, and arm mobility).

#### Session 13 (week 20): assessment tools/questionnaires and recording of cancer-specific medications and treatments of patients

At the last session (at week 20), the patient will receive via email a link (generated by REDCap) to open in a web browser the questionnaires to be filled in (Patient’s Last Session questionnaire, FACIT-F, PSQI, PHQ-9, GAD-7, BPI, NCCN Distress Thermometer, arm mobility, and PRFS). The study nurse will retrospectively verify after the patient’s last session (at week 20) whether there were any changes in the therapy during the intervention period (Medical History and Treatments questionnaire). The same procedure will be followed in case of withdrawal.

#### Six- and 12-month follow-up assessments (from week 20)

Finally, the patient will receive via email a link (generated by REDCap) to open in a web browser the questionnaires to be filled in at 6-month and 12-month follow-up (Patient’s Follow-up questionnaire, FACIT-F, PHQ-9, GAD-7, BPI, NCCN Distress Thermometer, arm mobility, and PRFS). After the 12-month follow-up assessment, the study nurse will fill in the questionnaire “End of Study”. The same procedure will be followed in case of withdrawal.

#### Reminders

If a patient has not filled in the questionnaires after 2 days, she will automatically receive a reminder via email each day for a period of 5 days. The therapist will also remind the patient to fill in the questionnaires if necessary (status “Incomplete/Partial/Completed Survey Response” visible in REDCap).

### Withdrawal and discontinuation

Patients can withdraw anytime from the study should they wish to do so. They will be invited to specify the reason for withdrawal and to fill in the questionnaire “end of study”. All data gathered until withdrawal will be included in the analysis, as laid out in the informed consent document.

The oncologist, as well as the eurythmy therapist or physical therapist, can decide at any time to exclude the patient from the study, e.g. in case of concern due to disease progression. If possible, patients will be asked to fill in the end of study questionnaire at the time of withdrawal.

### Reporting of adverse events

Evidence suggests that SAEs are not anticipated [40, 49, 75, 76]. Potential minor AEs that may be anticipated are dizziness, headache, and tension pain in the back and/or arms [75].

All SAEs will be documented and reported immediately (within a maximum of 24 h) to the sponsor. Both the investigator and the sponsor make a causality assessment of the event to the trial intervention. Any event assessed as possibly, probably, or definitely related is classified as related to the trial intervention. Both investigator and sponsor make a severity assessment of the event as mild, moderate, or severe. Mild means the complication is tolerable, moderate means it interferes with daily activities, and severe means it renders daily activities impossible. If it cannot be excluded that the SAE occurring is attributable to the intervention under investigation, the investigator will report it to the Ethics Committee within 15 days. The SAEs that are a clear result of the underlying disease (e.g., events occurring due to breast cancer or metastasis) are events that will not be reported. AEs and SAEs that are ongoing at study end are followed up until resolution or stabilization.

An annual safety report is submitted once a year to the Ethics Committee by the sponsor.

### Quality control and data protection

#### Quality measures

For quality assurance, the sponsor, the Ethics Committee, or an independent trial monitor may visit the research sites. Direct access to the source data and all study-related files is granted on such occasions. All involved parties keep the participant data strictly confidential.

#### Data handling and record keeping/archiving/data management system (hardware and software)

The CRFs in this trial are implemented electronically using a dedicated electronic data capturing (EDC) system (REDCap, https://www.project-redcap.org/). The EDC system is activated for the trial only after successfully passing a formal test procedure. All data entered in the CRFs are stored on a Linux server in a dedicated mySQL database. Responsibility for hosting the EDC system and the database lies with CTU Bern.

Questionnaires for patients will be prepared in electronic format and will be accessible through a personal link. The personal link will be sent to the patient’s email address. Patients’ email address will be therefore collected into REDCap but this field will be tagged as “Identifier field” and thus will be automatically removed from data exports. Patient’s diaries will be prepared in paper format and handed out by the study nurse at the site. The patient’s diary will be handed out with a prepared envelope (addressed and stamped) for sending it back to the sponsor. Date entry from paper diaries into REDCap will be done by the sponsor. The diaries will not contain any patient-identifying information.

#### Electronic and central data validation

Data is checked by the EDC system for completeness and plausibility. Furthermore, selected data points are cross-checked for plausibility with previously entered data for that participant. In addition, central data reviews will be performed by the sponsor on a regular basis to ensure completeness of the data collected and the accuracy of the primary outcome data. Before the database locks, the principal investigator in each center will validate the collected data with his signature.

#### Source documents

Source documents must be available at the site to document the existence of the study participant and must include the original documents relating to the study, as well as the medical treatment and medical history of the participant.

Source documents are considered (list not conclusive):

- Preliminary consent form and informed consent form
- Electronic/paper version of completed patient questionnaires (Medical history and treatments questionnaires, Patient’s Baseline questionnaire, Therapist’s questionnaire, Patient’s Last Session questionnaire, Patient’s Follow-up questionnaire, End of Study questionnaire, FACIT-F, PSQI, PHQ-9, GAD-7, BPI, NCCN Distress Thermometer, mobility of the arm, PRFS)
- Patient’s diary

#### Confidentiality and coding

Trial and participant data will be handled with uttermost discretion and is only accessible to authorized personnel who require the data to fulfill their duties within the scope of the study. On the CRFs and other study-specific documents, participants are only identified by a unique participant number. The names of the patients will not be disclosed. The participant number list will be stored at the Institute of Complementary and Integrative Medicine.

The server hosting the EDC system and the database is kept in a locked server room. Only the system administrators have direct access to the server and back-up tapes. A role concept with personal passwords (site investigator, statistician, monitor, administrator, etc.) regulates permission for each user to use the system and database as he/she requires.

All data entered into the CRFs are transferred to the database using Transport Layer Security (TLS) encryption. Each data point has attributes attached to it identifying the user who entered it with the exact time and date. Retrospective alterations of data in the database are recorded in an audit table. Time, table, data field and altered value, and the person are recorded (audit trail).

A multi-level back-up system is implemented. Back-ups of the whole system including the database are run internally several times per day and on external tapes once a day. The back-up tapes are stored in a secure place in a different building.

#### Retention and destruction of study data

At final analyses, data files will be extracted from the database into statistical packages to be analyzed. After database lock, the status of the database is recorded in special archive tables. The sponsor will keep the Trial Master File, the extracted data, the metadata, and interim/final reports for at least 10 years.

### Monitoring

Monitoring is performed by employee(s) of the sponsor who have received an introductory training on basic monitoring principles by CTU Bern. There is no Trial Steering Committee and no Stakeholder and Public Involvement Group (SPIG).

#### Site initiation visit

Site initiation visits are performed by the sponsor according to the Standard Operating Procedures (SOP) from CTU Bern.

#### Central data monitoring

Central data monitoring will be performed by the sponsor in order to ensure the overall completeness of data entry as well as plausibility and consistency of selected data points.

#### On-site monitoring visit(s)

No regular on-site monitoring visits will be performed for this study. Instead, monitoring visits are planned following a risk-based approach, with monitoring visits scheduled according to defined key performance indicators which will be checked at regular time points (ca. quarterly). However, sites may also be selected for a monitoring visit at random or whenever it is deemed necessary/appropriate by the sponsor at any time during trial conduct.

Any findings and comments during the monitoring visits will be documented in site visit reports and communicated to the local investigator and the sponsor. All source data and relevant documents will be accessible to monitors and questions to monitors are answered during site visits.

### Protocol amendments

The sponsor manages submission to the local Ethics Committees. Site personnel should be trained in the course of the study on any protocol amendment and any re-training should be provided as necessary. Self-training by site personnel may be acceptable, e.g., for non-substantial amendments. The local principal investigator is responsible that his/her site personnel is properly trained.

### Sample size calculation and statistical analysis

#### Sample size

Eurythmy therapy is expected to have a greater effect on CRF than the control intervention. A validated Minimal Clinically Important Difference (MCID) for FACIT-F subscale is defined as a change in score of 3–4 points [62]. We assume a non-parallel course over time of the FACIT-F subscale score between the ERYT group and the control group and a difference ≥ 4 in the FACIT-F subscale score between the two groups at the end of the intervention (week 20).

The mean FACIT-F subscale score at baseline for the two groups was assumed to be 30 points with a standard deviation of 10 points [60]. Simulations confirmed that the sample size of *N* = 156 patients (excluding drop-outs) will provide enough power (≥ 80%) to reject the null-hypothesis of no interaction between the group and time at the *α* = 0.05 level, if the effect of the ERYT intervention on the FACIT-F subscale score at the end of therapy is 4 points, if the standard deviation of the FACIT-F subscale score is ≤ 12 points, and if the within-patient variation between two measurements is ≤ 8 points. The simulations also confirmed that the power of 80% is reached in case of slight deviations from the assumptions if the difference between groups ≥ 4 points at the end of the intervention. To account for a dropout rate of 20%, the total sample size will be *N* = 196 patients (98 per group).

In order to ensure the study population can comply with the intervention as excepted, we will check feasibility issues, i.e., the number of sessions completed and compliance with home training, after the inclusion of 50 patients.

#### Statistical analysis

A nonparametric ANOVA model for longitudinal data, the F1-LD-F1 model of Brunner, Domhof, and Langer, will be applied to model the course of the FACIT-F subscale scores from baseline over the whole intervention [77]. The first F1 means that there is one group factor, the second that there is one-time or repeated measures factor. The main hypotheses are the following:

H0: there is no interaction of the treatment arm with the time;

H1: there is an interaction of the treatment arm with the time (favoring ERYT).

Least-square means of the original data (original scales) and 95% confidence intervals will be calculated and displayed in figures over time.

Differences from baseline to end of intervention will be analyzed by stratified Wilcoxon rank-sum tests (via ordinal logistic regression, i.e., proportional odds model with baseline measurements as co-variable, treatment arm as binary predictor and differences as outcome variable). Least-squares means of the original differences and 95% confidence intervals will be calculated.

Exploratory endpoints will be analyzed descriptively using mean and standard deviation or median and interquartile, as appropriate.

Statistical analysis will be conducted applying an intention-to-treat approach, and therefore including all randomized patients. In case of protocol non-adherence, a per-protocol analysis will be conducted to estimate the robustness of the primary estimates. All tests will be two-sided with a significance level of 0.05. Statistical analysis will be performed using R [78].

### Dissemination

Findings from the study will be published in peer-reviewed scientific journals, preferably in those with an open-access approach. Furthermore, results will be presented at international conferences on the subject of matter.

## Discussion

To our knowledge, this study is the first randomized clinical trial assessing eurythmy therapy as treatment for fatigue in metastatic breast cancer patients. Currently, there is no gold standard for the treatment of cancer-related fatigue. Physical activity can reduce CRF and is recommended during and after cancer treatment. However, it may be too burdensome for patients with metastatic breast cancer.

The first challenge we faced in designing this study was the choice of the control group. In order to control for unintended effects such as the therapist-patient interaction and active engagement (participation in a program), an active control intervention has been set up. The exercise specialist from our team developed the CoordiFit program, including similar movements as in ERYT but without mindfulness features. A second challenge was the data collection procedures. As patients have no hospital visits scheduled as part of the study during the intervention and follow-up periods, all questionnaires are sent via email to the patients. The clinical trial management system REDCap allows emails, including reminders, to be sent automatically to the patients. The physical and eurythmy therapists will also remind the patient to fill in the questionnaires.

Given that the success of eurythmy therapy depends on the regularity of the home training/compliance with the home training, participants are asked to record their exercises in a pre-printed patient diary during the whole intervention period. Studies generally report good compliance and satisfaction of participants with eurythmy therapy [40, 50].

Fatigue and major confounding factors/effect modifiers are assessed using validated questionnaires (e.g., FACIT-F for fatigue and quality of life, PSQI for sleeping disturbances, BPI for pain, GAD-7 for anxiety, PHQ-9 for depression, and NCCN Distress Thermometer for patient’s distress). In order to limit the number of questionnaires, social support is assessed by the subscale social/family well-being from FACIT-F. Since the intervention might also have a beneficial effect on the major confounding factors, these confounding factors are evaluated as secondary endpoint variables as well. All potential confounding factors including the patient’s socio-demographic data, time since diagnosis, stage of disease, initial metastatic breast cancer or later development, previous and current cancer therapy, co-morbidities which may cause CRF, and level of physical activity (at least once a week) will be taken into account for the evaluation of the effect of intervention on CRF.

Finally, advanced cancer populations are under-represented in clinical trials with non-pharmacological interventions. Given the distress that CRF causes patients, it is important to validate treatment options. This trial is a low-risk study and constitutes a novel approach to treat CRF. The expected results of the proposed research will be significant for several stakeholders.

- For patients, if eurythmy therapy is proven to be an effective therapeutic option, it will improve their fatigue, quality of life, and everyday life.
- For clinicians, if eurythmy therapy is proven to be beneficial, it will contribute to optimizing therapy for patients with CRF. Additionally, since CRF also constitutes a dose-limiting side effect for chemotherapy, improving CRF with eurythmy therapy may enable higher dosages or longer continuation of chemotherapy, which in turn may lead to a higher effectiveness and improve the outcome of chemotherapy.
- From a scientific point of view, given that no satisfactory therapy yet exists for CRF, further research to advance therapy including novel treatment approaches is highly desirable. This trial could pave the way for a safe and beneficial treatment option as well as future studies.
- Funding bodies could distinguish themselves by supporting scientific investigations of novel approaches in the treatment of CRF.

### Trial status

The overall study duration is 4 years, which includes a planned recruitment period of 36 months. The study duration for each patient is 20 weeks plus 2 follow-up visits at 6 and 12 months after the end of the intervention. The estimated study start date is March 2020. Estimated completion of recruitment is March 2022.

The protocol has been approved by the Ethics Committee of the Canton Bern. Due to the legal regulation in Switzerland, the usual procedure is to, firstly, submit and obtain approval of the Ethics Committee of a distinct Swiss Canton as a single center trial (University Hospital Bern). If that approval is obtained, participation of other centers and centers in other cantons will be later registered prior to their participation in the trial. Therefore, after we obtained the approval from the Ethics Committee, we translated all documents for the patients into French and Italian such that centers from all language regions Switzerland can participate. Afterwards, we have submitted an amendment that modifies the study as a multi-center trial, including the new documents in French and Italian. The amendment is currently under review by the respective Ethics Committee offices.

Protocol version number and date: version 2-1 (dated December 16, 2019).

## Data Availability

Not applicable.

## Abbreviations

ANOVA: Analysis of variance
BPI: Brief Pain Inventory
CTU: Clinical Trial Unit
ECOG: Eastern Cooperative Oncology Group
ERYT: Eurythmy therapy
FACIT-F: Functional Assessment of Chronic Illness Therapy – Fatigue
FACT-G: Functional Assessment of Cancer Therapy – General
GAD: General anxiety disorder
HRV: Heart rate variability
MCID: Minimally clinically important difference
NCCN: National Comprehensive Cancer Network
PG-SGA: Patient-Generated Subjective Global Assessment
PHQ: Patient Health Questionnaire
PRFS: Patient-Reported Functional Status
PSQI: Pittsburgh Sleep Quality Index
SAE: Severe adverse effect
